# Identification of potential TNF-α inhibitors: from in silico to in vitro studies

**DOI:** 10.1038/s41598-020-77750-3

**Published:** 2020-12-01

**Authors:** Komal Zia, Sajda Ashraf, Almas Jabeen, Maria Saeed, Mohammad Nur-e-Alam, Sarfaraz Ahmed, Adnan J. Al-Rehaily, Zaheer Ul-Haq

**Affiliations:** 1 Dr. Panjwani Center for Molecular Medicine and Drug Research, International Center for Chemical and Biological Sciences, University of Karachi, Karachi, 75270 Pakistan; 2 Department of Pharmacognosy, College of Pharmacy, King Saud University, P.O. Box. 2457, Riyadh, 11451 Kingdom of Saudi Arabia

**Keywords:** Computational biology and bioinformatics, Drug discovery

## Abstract

Tumor Necrosis Factor Alpha (TNF-α) is a pleiotropic pro-inflammatory cytokine. It act as central biological regulator in critical immune functions, but its dysregulation has been linked with a number of diseases. Inhibition of TNF-α has considerable therapeutic potential for diseases such as cancer, diabetes, and especially autoimmune diseases. Despite the fact that many small molecule inhibitors have been identified against TNF-α, no orally active drug has been reported yet which demand an urgent need of a small molecule drug against TNF-α. This study focuses on the development of ligand-based selective pharmacophore model to perform virtual screening of plant origin natural product database for the identification of potential inhibitors against TNF-α. The resultant hits, identified as actives were evaluated by molecular docking studies to get insight into their potential binding interaction with the target protein. Based on pharmacophore matching, interacting residues, docking score, more affinity towards TNF-α with diverse scaffolds five compounds were selected for in vitro activity study. Experimental validation led to the identification of three chemically diverse potential compounds with the IC_50_ 32.5 ± 4.5 µM, 6.5 ± 0.8 µM and 27.4 ± 1.7 µM, respectively.

## Introduction

Tumor necrosis factor is a pleiotropic pro-inflammatory cytokine, having effects on immune cells and plays a key role in cell proliferation, cell metabolism, inflammation, cell differentiation and apoptosis^1^. There are two types of TNF reported: tumor necrosis factor alpha (TNF-α) and tumor necrosis factor beta (TNF-β). Both are structurally and sequentially related to each other and compete for binding to their receptor^2^. TNF-α initiate many biological signaling pathways upon binding with two specific receptors, tumor necrosis factor receptor type I (TNFR1 or p55) and tumor necrosis factor receptor type II (TNFR2 or p75). Under normal physiological environment expression of TNFR2 is limited to immune cells, endothelial cells and nerve cells while TNFR1 expressed on almost all nucleated cells and play a key role in triggering the TNF-α signaling pathways^3^. Upon activation with binding to receptors, TNF-α initiate many major signaling pathways such as, activation of c-Jun N-terminal kinase, mitogen activated protein kinase (MAPK), extracellular signal regulated kinase (ERK) and transcription factor NFκB^4^. Physiologically it is an important regulator of immune functions, but its dysregulation has been linked with cancer, neurological diseases and, especially autoimmune diseases^5^.

Many TNF-α antagonists, icluding Infliximab, Certolizumab, Etanercept, Golimumab, and Adalimumab have revolutionized for therapeutic management of different autoimmune diseases, notably rheumatologic inflammatory diseases^6–10^. These drugs bind to the interface of TNF-α dimer and for a complex, which hinders the binding of TNF-α receptors thus stops the activation of downstream signaling complexes that induce inflammation and other signaling pathways. Drugs reported till to date are proteins or antibodies, with higher molecular weight and are associated with various side effects for instance, tuberculosis, congestive heart failure, lupus, demyelinating disease, injection site reaction, production of auto-antibodies and systematic side effects^11–14^. In 2005 He et al.^21^ reported the crystal structure of TNF-α dimer in complex with small molecule SPD304. The active site of TNF alpha at the interface of dimer consists of sixteen contact residues, including six tyrosine residues: Leu57, Tyr59, Ser60, Gln61, Tyr119, Leu120, Gly121, Gly122 and Tyr151 from chain A while Leu57, Tyr59, Ser60, Tyr119, Leu120, Gly121 and Tyr151 from chain B. Tyr119 is the most crucial residue, its chi-1 angles rotates to form a dimer by accommodating compound binding. Interaction of SPD304 with crucial residues slightly changed the symmetry of trimer, promoting the dissociation of monomer and stabilize the TNF-α dimer. This structure provide the direction for the development of small molecule inhibitors that can act as direct inhibitors of TNF-α by the stabilizing the dimer structure. To best of our knowledge no orally active small molecule drug is reported yet against TNF-α. Therefore, the identification of small molecules that can inhibit TNF-α regulated pathway presents a promising and current focus area.

Thus, in the present study, an integrated virtual screening approach was employed to explore new TNF-α inhibitors. The hits selected through virtual screening were then corroborated by using an in vitro assay. On the basis of TNF-α inhibition activity, compound 1, 2 and 3 showed the most promising inhibitory activity with the IC_50_ 32.5 ± 4.5 µM, 6.5 ± 0.8 µM and 27.4 ± 1.7 µM, respectively.

## Experimental

### Ligands preparation

Twenty six reported biologically active inhibitors (IC_50_ in the range of 0.001–100 µm) against TNF-α were retrieved from the literature (Supplementary Table S1)^15–25^. All the ligands were sketched in MOE^26^ using builder module. Ligands were subjected to protonation and energy minimize using MMFF94 force field^27^. The decoys were generated by submitting the set of active compounds to DUD-E website (http://dude.docking.org). For virtual screening, our In-house database of natural and synthetic molecules was utilized containing ~ 10,000 compounds. The database converted into the 3D format followed by protonation, structure correction and minimization using MOE^26^.

### Pharmacophore-based virtual screening

In silico pharmacophore-based virtual screening is a sophisticated tool in the modern drug discovery process for the identification of new leads from large database with the desired activity profile^28^. The availability of large compounds library of TNF-α inhibitors and their corresponding biological activity on different cell lines have enabled us to focus on ligand-based pharmacophore modelling approach.

In this approach, conformations of selective active compounds are aligned, and common pharmacophore features are generated where physicochemical functionalities are overlapped. These conformations were calculated from OMEGA, implemented in LigandScout 4.3^29^. The “create shared pharmacophore” function of LigandScout was used to create 12 hypothesis of pharmacophore models comprising diverse chemical features (Hydrogen bond donor, Aromatic, Hydrophobic, Hydrogen bond acceptor and many exclusion volume). Table 1 represented the generated hypothesis models using different combinations of shared features of active compounds.

**Table 1:** Generated hypothesis of pharmacophore models and their shared features with the hit rate of active, inactive and decoy compounds after screening.

S. no	Hypothesis	Shared features	Actives hit rate %	Inactives hit rate %	Decoys hit rate %
1	Hypo_1	Hyd, Hyd, Hyd, HBA	85	33	55
2	Hypo_2	Hyd, Hyd, Hyd, Ar, HBA, HBA	93	33	50
3	Hypo_3	Hyd, Hyd, Hyd, Ar, HBA, HBA	75	50	27
4	Hypo_4	Hyd, Hyd, Ar, HBA, HBA	28	16	22
5	Hypo_5	Hyd, Hyd, Hyd, HBD, HBA, HBA	77	16	27
6	Hypo_6	Hyd, Hyd, Hyd, Ar, HBA, HBA	93	33	50
7	Hypo_7	Hyd, Hyd, Hyd, HBA, HBA	73	16	6
8	Hypo_8	Hyd, Hyd, Hyd, HBA,	85	33	55
9	Hypo_9	Hyd, Hyd, Ar, HBA,	10	0	3
10	Hypo_10	Hyd, Hyd, HBD, HBA	40	33	25
11	Hypo_11	Hyd, Hyd, HBD, HBA	40	33	25
12	Hypo_12	Hyd, Ar, HBA, HBD	3	0	0

The generated pharmacophore model was validated in order to examine its potential to differentiate between active and decoys compounds in the dataset. For this purpose, an external test set was prepared using 26 confirmed actives and 1750 decoys or inactive for the validation of pharmacophore hypothesis.

Various statistical parameters such as percent ratio of actives, percentage yield of actives, false negatives, false positives, enrichment factor (EF), and area under the ROC curve (AUC) were further calculated.

The validated pharmacophore model was used as a 3D query to retrieve the potential compounds from the inhouse natural product and synthetic compounds library (~ 10,000 compounds). Default settings were used for virtual screening (25 conformers generated per entry under omega-fast settings). The resultant compounds were subjected to drug-like assessment employing the ADMET Descriptors and Filter by Lipinski rule. The compounds that have obeyed these criteria were upgraded to the molecular docking studies.

### Molecular docking

A campaign of high throughput docking was performed by using MGLTools of AutoDock 4.2. The crystal structure of TNF-α with PDB ID 2AZ5^21^ was retrieved from Protein Data Bank. Missing residues in the crystal structure were added and refined by using MODELLER 9.18^30^. Polar hydrogen atoms were added while nonpolar were merged and Gasteiger charges were applied. Lamarckian genetic algorithm was used for all the calculations. The coordinates of the crystal structure was saved into pdbqt format for docking calculation. A grid box of centers 28 Å × 26 Å × 30 Å along with dimensions − 19.515 Å × 74.84 Å × 33.894 Å, centered on cognate ligand served as spacing grid which encompassed the entire binding site. The pose with the highest binding affinity and reasonable able conformation were chosen for further analysis. The finger prints of protein–ligand interactions were analyzed by using PLIF module in MOE. Furthermore, the compounds were visually analyzed by using Chimera^31^.

### In vitro studies

THP-1 cells (Human monocytic leukemia cells) were obtained from Biobank facility, ICCBS, University of Karachi, which was purchased from the ECACC (European Collection of Cell Cultures, UK). The cells were cultured in RPMI-1640, supplemented with 10% FBS (fetal bovine serum), 50 µmol/L mercaptoethanol (Merck, Damstadt, Germany), 5.5 mmol/L glucose (BioM Laboratories, Chemical Division, Malaysia), 2 mmol/L; l-glutamine (PAA Laboratories, GmbH, Pasching, Austria), 10 mmol/L HEPES (MP Biomedicals, lLLKIRCH, France) and 1 mmol/L sodium pyruvate (GIBCO, Grand Island, NY, USA). Cells were harvested and 2 × 10^5^ cells/mL were then added to 24-well tissue culture plates. Differentiation of cells was performed by adding 20 ng/mL of phorbol myristate acetate (PMA) (SERVA, Heidelberg, Germany), and by incubation at 37 °C for 24 h in 5% CO_2_. Cells were then activated with 50 ng/mL of *E. coli* lipo-polysacchride B (DIFCO Laboratories, USA), and treated with different concentration of test compounds (1, 10, and 100 µg/mL) and, were then incubated at 37 °C for 4 h in 5% CO. The supernatants were analyzed for the level of TNF-α using Human TNF-α ELISA Kit (R&D Systems, Minneapolis, USA).

Briefly the working concentration of 4 μg/mL in PBS from 720 μg/mL of mouse anti-human TNF-α capture antibodies were used for coating of 96 well ELISA plate. The 100 μl of TNF capture antibody per well was added and plate was incubated overnight at RT. The plate was then blocked by adding 300 μl of reagent diluent in each well and incubated for 1 h at RT. 100 μL/well of collected supernatants was then added each in triplicate and plate was then incubated for 2 h at RT. The 100 μL of detection antibody diluted to 250 ng/mL (working concentration) in reagent diluent was then added to each well and plate was incubated for 2 h at RT. Next 100 μL of 1:200 dilution of streptavidin-HRP in reagent diluent was added in each well in dark and incubated for 20 min at RT. Substrate solution was prepared by mixing color reagent A and color reagent B (provided in the kit) in 1:1 ratio and 100 μL from this mixture was added to each well in dark, plate was incubated for 20 min at RT. The reaction was stopped by adding 50 μL of stop solution (2 N H_2_SO_4_) and plate was then read at wavelength of 450 nm in ELISA reader (ELX800 NB, DIA LAB, Wr. Neudrof, Austria).

### MTT cytotoxicity assay

Cytotoxicity of compounds on NIH-3T3 fibroblast cells was evaluated by MTT colorimetric assay. The cell line was provided by ICCBS Biobank facility which was purchased from (ATCC, Manassas, USA). Briefly 100 μL of 6 × 10^4^ cells/mL in DMEM supplemented with 10% FBS were plated into 96-wells flat bottom plate and incubated overnight at 37 ºC in 5% CO2. Different concentrations of test compounds (350–1 µM) were added to the plate in triplicates and incubated for 48 h. 50 µL of 0.5 mg/mL MTT was added to each well and plate was then further incubated for 4 h. MTT was aspirated and 100 µL of DMSO was then added to each well. The extent of MTT reduction to formazan within cells was calculated by measuring the absorbance at 540 nm, using spectrophotometer (Spectra Max plus, Molecular Devices, CA, USA). The cytotoxic activity was recorded as concentration causing 50% growth inhibition (IC_50_) for 3T3 cells.

## Results and discussion

### Pharmacophore-based virtual screening

**Figure 1:**
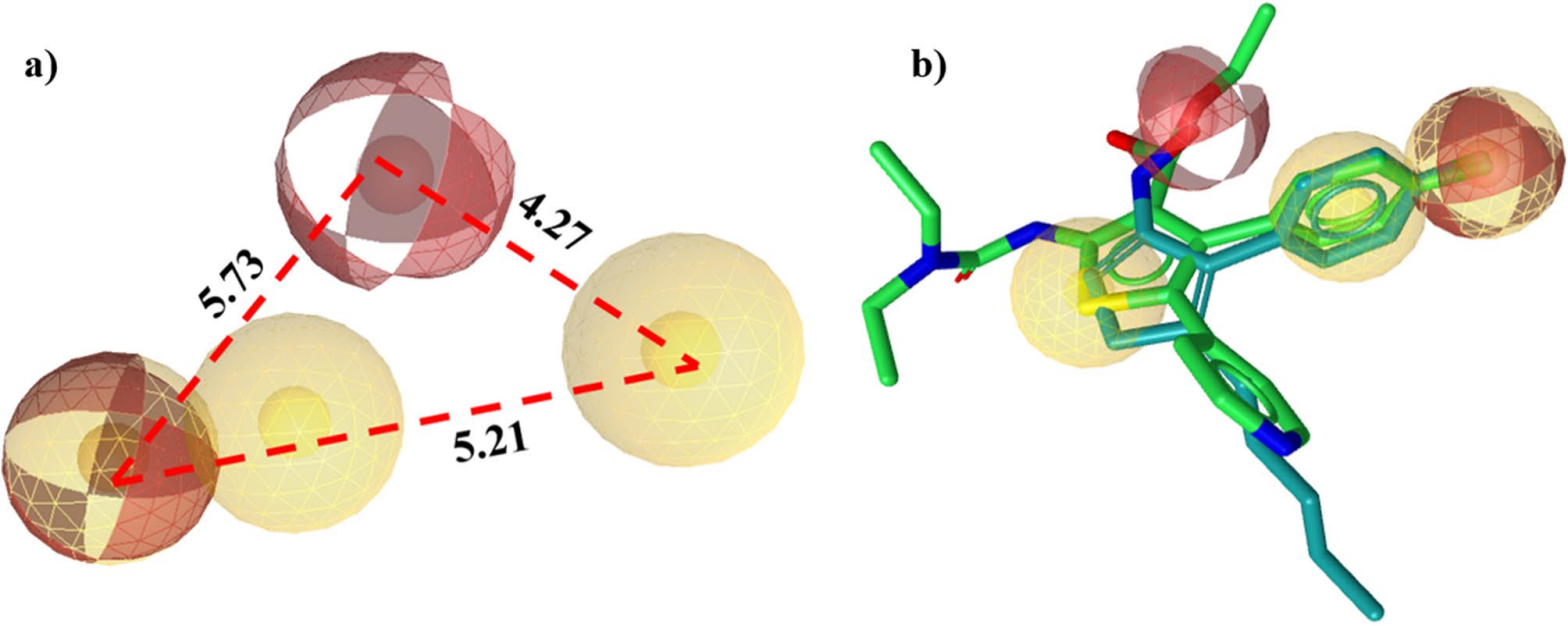
(**a**) Chemical features of best pharmacophore hypothesis (Hypo_7) with their inter-feature distance constraints in angstrom (Å). (**b**) Two active inhibitors of TNF-α from which final pharmacophore model was generated, aligned on Hypo_7. Hydrophobic feature indicated as yellow sphere while hydrogen bond acceptor as red sphere.

Prior to the pharmacophore model generation, key features of the reported 28 active compounds from 10 different classes (pyrazolones, urea, indole, thiophene, purine, oxime, diaryl heptanoids etc.) were identified by superposing them to determine potential overlapped chemical features with the LigandScout. This procedure has generated 12 hypothesis (Table 1) with three to six potential chemical features. For the selection and validation of the best model, these generated hypotheses were refined and pruned on the basis of the following criteria:

1. the presence of chemical features that possibly interact with tyrosine residues (potentially Tyr119), which is crucial for TNF-α inhibition.
2. ability to select active compounds with good fitness score according to their biological activity with minimum deviation and.
3. ability to picked active compounds from the pool of active and decoys dataset. This criteria declare Hypo_7 as best hypothesis as it yields the pharmacophore fit score range that imitate the activity trend and difference in their magnitude as illustrated in Table 1. The Hypo_7 was in good agreement according to the nature of TNF-α active site residues as it contain three hydrophobic features for the key interaction with Leu57, Tyr59, Tyr119 and Tyr151 and two hydrogen bond acceptor for the interaction with Ser60 and Gln61 (Fig. 1a). Moreover, Hypo_7 align well on two of the highly active TNF-α inhibitors (Fig. 1b). Further the quality of selected model was determined by calculating the enrichment factor i.e. the fraction of actives compounds within a database while the values of other parameters illustrated in Table 2;

$${\text{EF}} = {\text{D}} \times {\text{Ha/A}} \times {\text{Ht}}$$

where D is the total number of molecules in the decoy dataset, A is the total number of active compounds, Ha is the number of active hits molecules while Ht is the number of decoy hits molecules. The enrichment factor for the Hypo_7 was found to 12.5. Moreover, ROC curve was also calculated to measure the sensitivity (ability to search true positives) and specificity (ability to avoid false positives) between active and decoys on the basis of pharmacophore fitness score and the value of AUC was found to be 0.83 (Supplementary Fig. S1).

**Table 2:** Statistical parameters of enrichment factor for Hypo_7 model.

Parameters	No. of compounds
Total no. of decoy compounds (D)	1750
Total no. of TNF-α active inhibitors (A)	28
No. of decoys hits (Ht)	100
No. of active hits (Ha)	20

**Figure 2:**
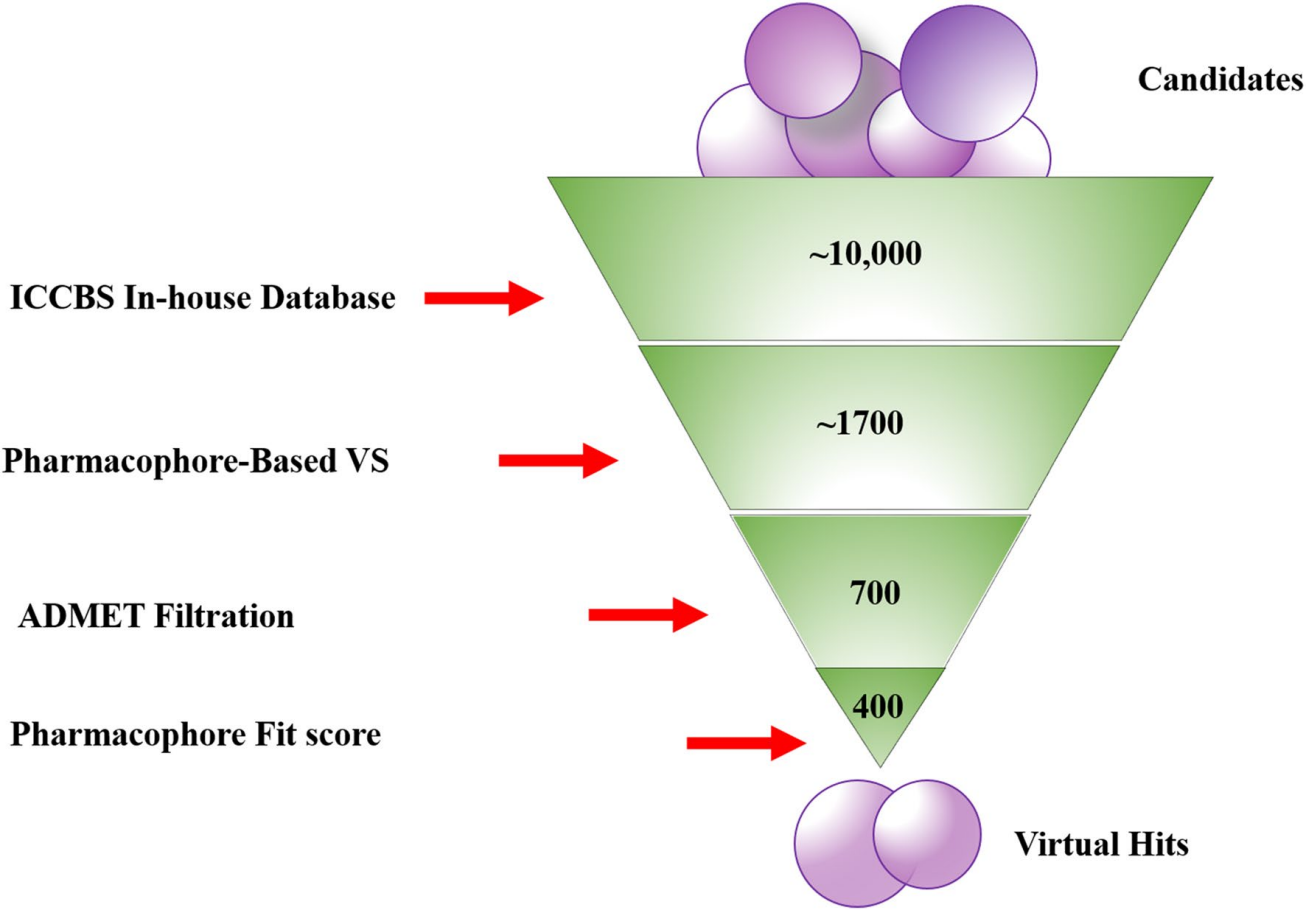
Schematic representation of pharmacophore-based virtual screening.

For virtual screening in-house database of ICCBS containing ~ 10,000 compounds were virtually screened against the final pharmacophore model. In the result of successive screening ~ 1700 compounds were obtained. Drug like filter was applied on the database: molecular weight < 500 Dalton, no. of H bond acceptor < 5, no. of H bond donor < 10, no. of rotatable bond < 11 and octanol water partition coefficient log *p* < 5. Assessment of ADMET filtration led to the identification of 700 hit compounds for further analysis. The retrieved hits were further reduced to 400 compounds based on highest pharmacophore fit score in the range of 58.5–55.5 and subjected to molecular docking studies. The overall workflow of pharmacophore-based screening shown in Fig. 2.

### Molecular docking studies

The binding mode interactions of hits, obtained from pharmacophore based screening, were investigated by molecular docking studies. All the hits were docked in the active site of the target protein within the define grid. The top ranked 142 compounds with the cut off score of  > − 6.5 kcal/mol were selected for further analysis. Extracted compounds were subjected to protein–ligand interactions fingerprinting by using PLIF module implemented in MOE. Interaction pattern of 142 compounds were analyzed which led us to the identification of five virtual hits from three different chemical classes. Upon investigating the binding mode, it was revealed that the identified hits reside with the same pattern in the active site of TNF-α as the co-crystallized ligand (SPD304). This suggested that the virtual hits might act in similar way as reference compound SPD304 inhibit the protein activity.

**Figure 3:**
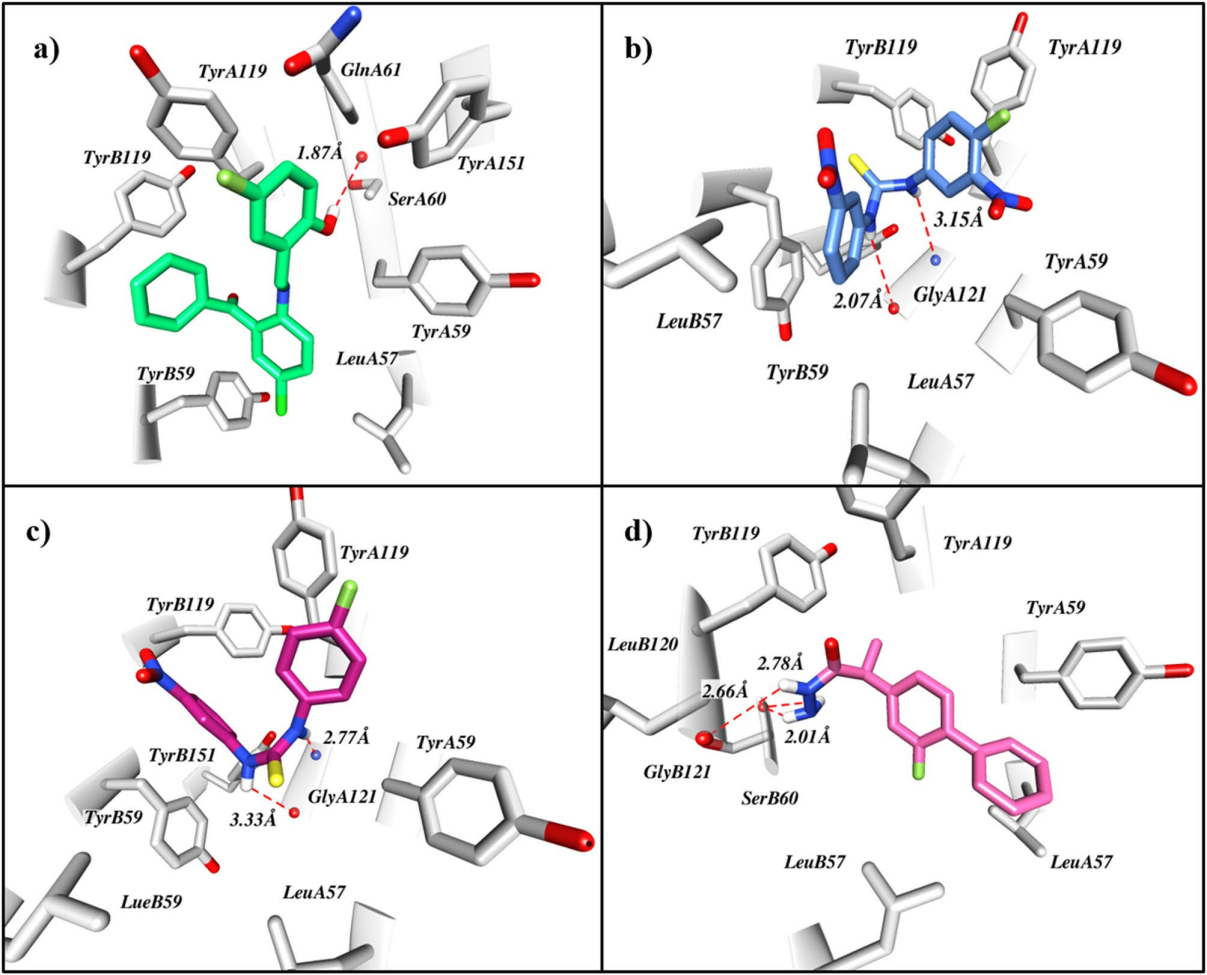
Docked pose of virtual hits in the active site of TNF-α. (**a**) Compound 1, benzophenone derivative establishes several hydrophobic interactions with crucial residues. (**b**,**c**) Compound 2 and 3, thiourea derivatives around the hydrophobic cleft formed by active site residues. (**d**) Compound 5, flurbiprofen derivative stabilize the TNF-α by making many hydrophobic and three hydrogen bonds with the active site residues. Compounds are shown in different color while protein residues shown in light grey color. Red dash lines represented the hydrogen bond contacts.

Compound 1 showed good inhibitory potential against TNF-α with the binding affinity of − 8.4 kcal/mol. Detailed molecular interaction pattern of compound 1 demonstrated that chlorobenzene ring, establish π–π stacking interaction with the aromatic ring of TyrB59 and TyrB119 while π-alkyl interaction with LeuA57 and TyrA59. Similarly, fluorobenzene ring involve in mediating π-alkyl interaction with TyrA59, GlnA61, TyrA119, and TyrA151. Moreover protein–ligand contacts were stabilized by two hydrogen bond interactions between hydroxyl group of the ligand and the side chain of SerA60 (Fig. 3a).

Compound 2, 3 and 4 were belong to chemical class of thiourea with binding affinities − 7.1, − 7.0 and − 6.6 kcal/mol respectively. Compound 2 and 3 were more active as compared to compound 4 based on TNF-α inhibition activity. Upon evaluating the molecular interactions, it was suggested the compound 2 and 3 showed similar type of interactions (Fig. 3b,c). The hydrophobic cleft formed by the Leu59 and Tyr59, Tyr119 and Tyr151 around both compounds provide additional stability to better fit the ligands in the active site of TNF-α. In case of compound 2, π-alkyl interactions was observed between nitrobenzene ring and LeuA57, LeuB57 and TyrB59 while other nitrobenzene ring exhibiting fluorine group showed π-alkyl interaction with TyrA119. Similarly, in case of compound 3 π-alkyl interaction observed between fluorobenzene ring and TyrA59 and TyrA119 while nitrobenzene ring in the ligand mediate π-alkyl interaction with TyrB59 and TyrB119. Moreover, both compounds observed to establish hydrogen bond contacts with GlyA121. In case of compound 4, any interaction with the hydrophobic cleft is not observed, which may account for the low potency of this compound.

Detailed binding mode analysis revealed that compound 5 mediated similar type of interaction as observed for SPD304 (Fig. 3d). Binding affinity of compound 5 with the TNF-alpha was found to be − 7.4 kcal/mol. Benzene ring of the ligand interact with the side chain of LeuA57, LeuB57 and TyrA59 by mediating π-alkyl interactions while methyl group of the ligand establish alkyl-alkyl interaction with TyrB119. Similarly, the nitrogen of hydrazine group in the ligand establish three hydrogen bond contacts with SerB60 and LeuB120. Four compounds among all the docked compounds showed strong hydrophobic interactions along with the hydrogen bond contacts with the crucial residues of the TNF-α which suggested that these compounds might be serve as direct inhibitors of the target protein.

### Bioassay validation

Compounds obtained from in silico studies were evaluated for their TNF-α inhibition activity through in vitro studies using Pentoxifyllin as standard drug. Table 3 list the TNF-α inhibitory activity for the standard and shortlisted compounds obtained from virtual screening. Shortlisted compounds were the derivatives of benzophenone, flurbiprofen and thiourea. Compound 1, a benzophenone derivative synthesized and reported by Arshia et al.^32^ from our institute*,* showed significant inhibition against TNF-α with the IC_50_ value of 32.5 ± 4.5 µM. Benzophenone molecule possesses good anti-inflammatory activity e.g. ketoprofen contain benzophenone group is one of the marketed anti-inflammatory drugs^33,34^. Compound 2–4 were the derivatives of thiourea, previously reported by Bilquees et al.^35^, from our institute all these compounds showed potent inhibition (6.5 ± 0.8, 27.4 ± 1.7 and 280.6 ± 9.6 µM respectively), except compound 4 as compare to standard pentoxifyllin (IC_50_ = 340.6 ± 7.54 µM), against TNF-α. Similarly compound 5 was the derivative of Flurbiprofen; the most important NSAIDs (non-steroidal anti-inflammatory drugs), which is widely used to treat arthritis^36^, previously synthesized by Momin et al.^37^, from our institute. Compound 5 showed inhibition against TNF-α with the IC_50_ value of 117.7 ± 1.1 µM. Moreover, all compounds were found to be non-toxic on NIH-3T3 cell line when compared to the standard drug cyclohexamide (Table 3). In vitro studies were in good agreement with in silico studies and revealed that all the tested compounds inhibit TNF-α produced from lipopolysaccharide (LPS) activated THP-1 cells, which explain the potencies of these compounds.

**Table 3:** Structure of newly identified virtual hits against TNF-α and their IC_50_ values. Data represent the mean ± SEM of triplicate determination.

S. no.	Compound ID	Structure of virtual hits	TNF-α inhibition (IC_50_ µM)	Cytotoxicity on NIH-3T3 cells (IC_50_ µM)
1	Compound 1		32.5 ± 4.5	43.2 ± 0.28
2	Compound 2		6.5 ± 0.8	204.04 ± 28.5
3	Compound 3		27.4 ± 1.7	84.7 ± 9.2
4	Compound 4		280.6 ± 9.6	95.0 ± 20.7
5	Compound 5		117.7 ± 1.1	104.95 ± 1.16
6	Pentoxifylline		340.6 ± 7.54	-
7	Cyclohexamide (standard drug cytotoxicity)		–	0.46 ± 0.07

## Conclusion

Inhibition of TNF-α has emerged as a potential therapeutic to treat tumor and especially autoimmune diseases. Currently, no orally active FDA approved drug against TNF-α is reported. Small-molecule drugs that can regulate TNF-α levels or activity may provide an economic alternative to antibody therapeutics. In the current investigation we aim to identify novel small molecule from inhouse database that obey the pharmacophoric features of TNF-α inhibitors. The statistical evaluation of the developed pharmacophore model highlights its ability to discriminate between active and decoys. The screened compounds were subjected to molecular docking to investigate their binding mode. Subsequently, 5 compounds were identified with high docking score and demonstrated key interaction as was noticed for reference compound. Experimental validation indicated that three of these compounds exhibit strong TNF-α inhibitory potential with the IC_50_ 32.5 ± 4.5 µM, 6.5 ± 0.8 µM and 27.4 ± 1.7 µM, respectively. The identified inhibitors have potential to act as anti-inflammatory agent and may serve as a starting point in developing novel drugs.

## Supplementary information

Supplementary Information.
