# Supplementary material for: Identification of potential TNF-α inhibitors: from in silico to in vitro studies
Source: Sci Rep. 2020 Dec 1;10:20974. doi: 10.1038/s41598-020-77750-3 (PMC7708426; doi:10.1038/s41598-020-77750-3)
Supplement: Supplementary file 1 — Supplementary Information. [file 41598_2020_77750_MOESM1_ESM.docx]

Identification of Potential TNF-α Inhibitors: From *In Silico* to *In Vitro* Studies

Komal Zia^1^, Sajda Ashraf^1^, Almas Jabeen^1^, Maria Saeed^1^ Mohammad Nur-e-Alam^2^, Sarfaraz Ahmed^2^ Adnan J. Al-Rehaily^2^ and [Zaheer Ul-Haq](http://www.sciencedirect.com/science?_ob=RedirectURL&_method=outwardLink&_partnerName=27983&_origin=article&_zone=art_page&_linkType=scopusAuthorDocuments&_targetURL=http%3A%2F%2Fwww.scopus.com%2Fscopus%2Finward%2Fauthor.url%3FpartnerID%3D10%26rel%3D3.0.0%26sortField%3Dcited%26sortOrder%3Dasc%26author%3DUl-Haq,%2520Zaheer%26authorID%3D26024448000%26md5%3De5674198b9eb31fab0cf0bacc139910d&_acct=C000060484&_version=1&_userid=3415223&md5=83d2f3a262a6b9a51c5e04ced2e28348)^1*^

**^1^Dr. Panjwani Center for Molecular Medicine and Drug Research, International Center for Chemical and Biological Sciences, University of Karachi, Karachi-75270, Pakistan.**

**^2^Department of Pharmacognosy, College of Pharmacy, King Saud University, P.O. Box. 2457, Riyadh 11451, Kingdom of Saudi Arabia**

[*Corresponding Author](mailto:Corresponding%20Author%20Zaheer%20Ul-Haq%20Dr.%20Panjwani%20Center%20for%20Molecular%20Medicine%20and%20Drug%20Research,International%20Center%20for%20Chemical%20and%20Biological%20Sciences,%20University%20of%20Karachi,%20Karachi-75270,%20Pakistan.Email:%20zaheer.qasmi@iccs.edu)

[**Zaheer Ul-Haq**](mailto:Corresponding%20Author%20Zaheer%20Ul-Haq%20Dr.%20Panjwani%20Center%20for%20Molecular%20Medicine%20and%20Drug%20Research,International%20Center%20for%20Chemical%20and%20Biological%20Sciences,%20University%20of%20Karachi,%20Karachi-75270,%20Pakistan.Email:%20zaheer.qasmi@iccs.edu)

[Dr. Panjwani Center for Molecular Medicine and Drug Research,](mailto:Corresponding%20Author%20Zaheer%20Ul-Haq%20Dr.%20Panjwani%20Center%20for%20Molecular%20Medicine%20and%20Drug%20Research,International%20Center%20for%20Chemical%20and%20Biological%20Sciences,%20University%20of%20Karachi,%20Karachi-75270,%20Pakistan.Email:%20zaheer.qasmi@iccs.edu)

[International Center for Chemical and Biological Sciences,](mailto:Corresponding%20Author%20Zaheer%20Ul-Haq%20Dr.%20Panjwani%20Center%20for%20Molecular%20Medicine%20and%20Drug%20Research,International%20Center%20for%20Chemical%20and%20Biological%20Sciences,%20University%20of%20Karachi,%20Karachi-75270,%20Pakistan.Email:%20zaheer.qasmi@iccs.edu)

[University of Karachi, Karachi-75270, Pakistan.](mailto:Corresponding%20Author%20Zaheer%20Ul-Haq%20Dr.%20Panjwani%20Center%20for%20Molecular%20Medicine%20and%20Drug%20Research,International%20Center%20for%20Chemical%20and%20Biological%20Sciences,%20University%20of%20Karachi,%20Karachi-75270,%20Pakistan.Email:%20zaheer.qasmi@iccs.edu)

[Email: zaheer.qasmi@iccs.edu](mailto:Corresponding%20Author%20Zaheer%20Ul-Haq%20Dr.%20Panjwani%20Center%20for%20Molecular%20Medicine%20and%20Drug%20Research,International%20Center%20for%20Chemical%20and%20Biological%20Sciences,%20University%20of%20Karachi,%20Karachi-75270,%20Pakistan.Email:%20zaheer.qasmi@iccs.edu)

Tel: +9221-111-222-292 Ext 309

Fax: +9221-34819018, 19

**Pharmacophore Generation and Validation**

A pharmacophore is an abstract description of molecular features which are necessary for recognition of a ligand by a biological macromolecule. Reported TNF-α inhibitors were utilized to build a pharmacophore model (Table S1). Twenty six actives, six inactives and 1750 decoys were used as testing set to validate the pharmacophore model. Initially, by the alignment of shared features of reported inhibitors with diverse scaffold 12 pharmacophore hypotheses were generated comprising of four different chemical features. These generated pharmacophore hypotheses by evaluated by the ability to pick most of the active compounds and align on them with highest fitness score. Among the generated hypotheses, Hypo_7 (generated by the shared features of compound 4 and 9) correctly picked the most of active compounds with the fitness score in the range of 58 to 55. To further validate the accuracy of Hypo_7, ROC curve was plotted. Receiver operating characteristic, or ROC curve, is a statistical method used in the validation of the pharmacophore model and method of virtual screening. ROC is appropriate in retrieving both information i.e. actives as well as in-actives and it is independent of the number of active compounds. Quantitative measure i.e. area under the curve (AUC) is used to evaluate the performance of the overall method and it ranges from zero to one. ROC curve was plotted between Sensitivity (True Positives) to the function of Specificity (False Positives) at different interval of thresholds (Figure S1). The ROC curve value for Hypo_7 was found to be 0.83, reveals that the selected pharmacophore model is good.

**Table S1.** Structure and reported IC_50_ of TNF-α inhibitors used in the construction and validation of pharmacophore model.

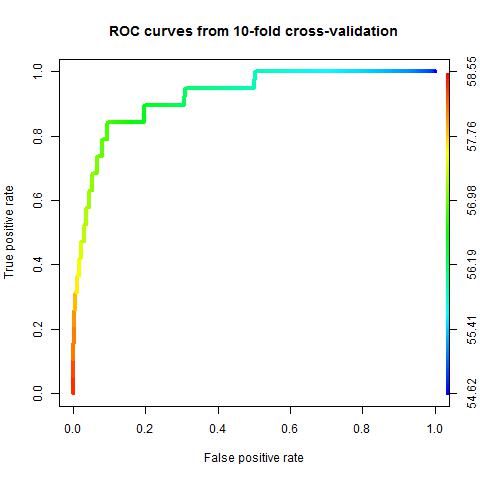


**Figure S1.** Graphical representation of ROC curve between true positive rate (selectivity) vs false positive rate (1-specificity).

**Re-docking Experiment**

The reproduction of co-crystallized pose of cognate ligand at the active site of the protein validates the reliability of docking protocol. To evaluate the docking accuracy of MOE and AutoDock 4.2., re-docking experiment was conducted by utilizing PDB ID 2AZ5. The cognate ligand was extracted, prepared and redock into the active site by using rigid receptor docking protocol. The analysis of redock pose reveal that AutoDock 4.2. possessed the high accuracy for predicting the RMSD (0.68 Å) (Figure S3) while MOE failed to reproduce the crystal pose with RMSD of 5.08 Å. Thus, AutoDock 4.2. was selected to performed the virtual screening of inhouse database for discovery of potent inhibitors against TNF-alpha.


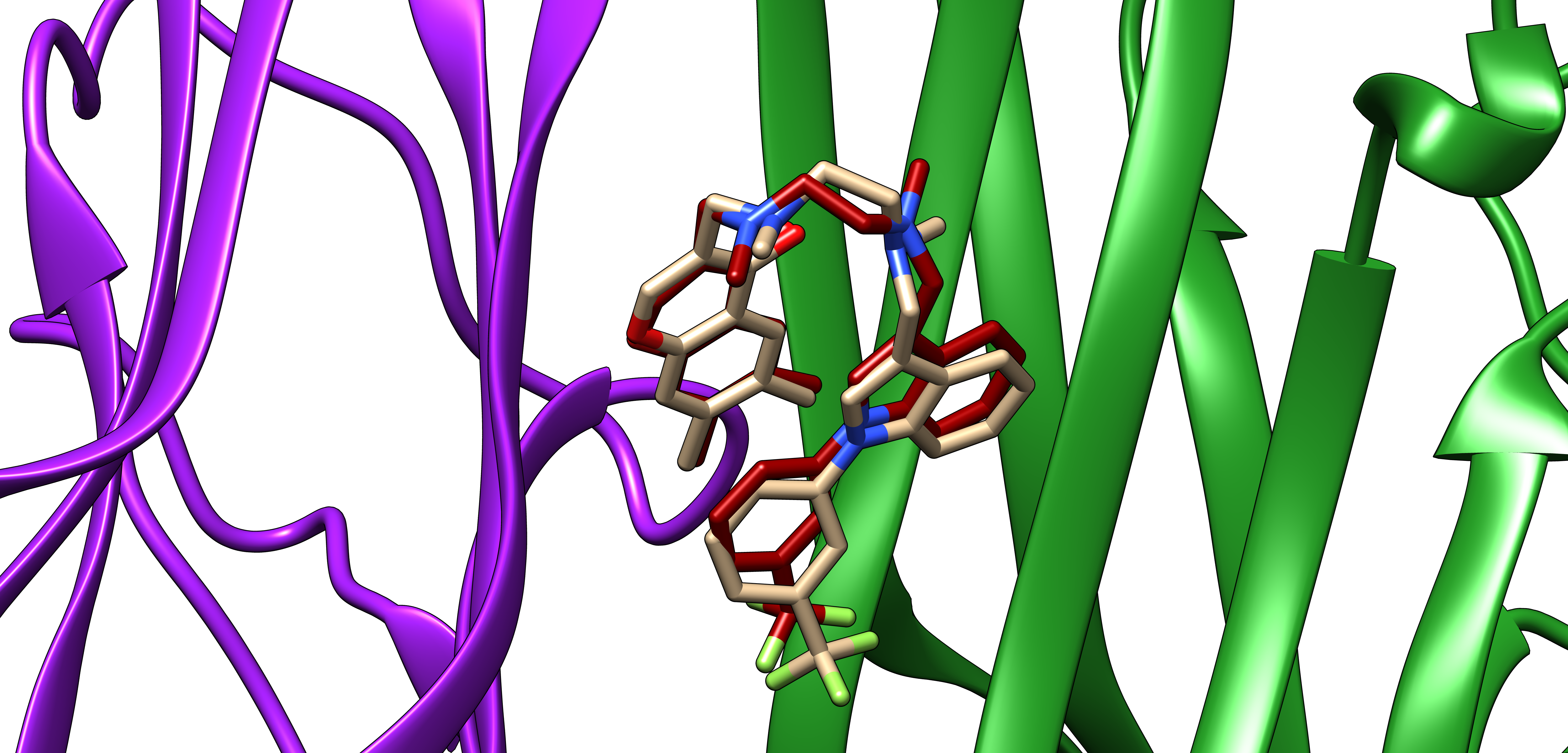


**Figure S2.** Redocking results showing the cognate ligand (fire brick) and the docked pose (tan) produced by AutoDock.
